# Supplementary material for: XIST Is a Key Modulator Associated With the Adhesome Network
Source: FASEB Bioadv. 2026 Sep 19;8(9):e70151. doi: 10.1096/fba.2026-00185 (PMC13589267; doi:10.1096/fba.2026-00185)
Supplement: Supplementary file 1 [file FBA2-8-e70151-s001.zip › fba270151-sup-0001-Figures.docx]

Supplementary Figures

**Figure S1.** (a-b) Volcano plots showing the differentially expressed adhesome genes after *XIST* knockdown in OVCAR3 clones X7 (a) and X9 (b). Adhesome genes with |log2FoldChange| > 3 or -log10(padj) > 20 were labeled with their gene names. (c-d) Counts of downregulated and upregulated adhesome genes classified as integrin-associated (Integrin), cadherin-associated (Cadherin), or shared between both categories (Both) in *XIST*-knockdown OVCAR3 clones X7 (c) and X9 (d); statistical comparisons were performed using a one-tailed binomial test. (e) Principal component analysis (PCA) of all nine samples, including control cells (Ctrl, n = 3) and two *XIST* knockdown clones (X7 and X9, n = 3 each), performed using DESeq2. (f) RT-qPCR analysis confirmed reduced expression of XIST and representative adhesome genes (CTNNB1, CTNND2, and LAYN) following XIST knockdown in OVCAR3 cells. Statistical significance was assessed using a one-way ANOVA test; ns, not significant; **, p < 0.01; ***, p < 0.001; ****, p < 0.0001.

**Figure S2.** (a-b) *Xist* expression levels (log_2_(TPM+0.001)) across tissue types in GTEx (a) and TCGA (b). (c-d) Distribution of median |log2FoldChange| values for the adhesome gene set and 2,000 randomly generated expression- and size-matched gene sets following *XIST* knockdown in OVCAR3 clones X7 (c) and X9 (d). The observed median |log2FoldChange| value of the adhesome gene set (red dashed line) exceeded those of all random permutations. The empirical p-value was calculated as the fraction of random gene sets whose median |log2FoldChange| values were greater than or equal to the observed median |log2FoldChange| of the adhesome gene set, using the formula (1 + k) / (1 + N), where k is the number of permutations ≥ the observed value and N is the total number of permutations.

**Figure S3.** (a-b) Correlations between lncRNAs and adhesome genes in female normal tissues (a) and female primary tumors (b); statistical significance was assessed using a one-tailed Wilcoxon test comparing *Xist* with each of the other lncRNAs. (c-d) Correlation between *XIST* and adhesome genes across tissue types in female normal tissues (c) and female primary tumors (d). (e) Correlations between lncRNAs and adhesome genes in mouse normal tissues; statistical significance was assessed using a one-tailed Wilcoxon test comparing *Xist* with each of the other lncRNAs. ns, not significant; **, p < 0.01; ****, p < 0.0001.
